# Genome-wide association study of Fuchs’ endothelial corneal dystrophy in the German population

**DOI:** 10.1007/s00439-025-02749-7

**Published:** 2025-05-12

**Authors:** Juliane Fechner, Guilherme B. Neumann, Fabia Murza, Leonard Matthias, Marcus Walckling, Claudia Brockmann, Thomas A. Fuchsluger, Tobias Brockmann

**Affiliations:** 1 Department of Ophthalmology, Universitätsmedizin Rostock, Doberaner Straße 140, 18057 Rostock, Germany; 2 Albrecht Daniel Thaer-Institute for Agricultural and Horticultural Sciences, Humboldt-Universität zu Berlin, Invalidenstraße 42, 10115 Berlin, Germany; 3 Department of Wildlife Diseases, Leibniz Institute for Zoo and Wildlife Research, Alfred-Kowalke-Straße 17, 10315 Berlin, Germany; 4 Department SciTec, Ernst-Abbe-Hochschule Jena, University of Applied Sciences Jena, Carl-Zeiss-Promenade 2, 07745 Jena, Germany

**Keywords:** Genome-Wide Association Study (GWAS), Fuchs Endothelial Corneal Dystrophy (FECD), TCF4, Long non-coding RNA (lncRNA), Genetics

## Abstract

The genetic etiology of Fuchs Endothelial Corneal Dystrophy (FECD) is not yet fully elucidated. While the disease is widespread and the leading indication for corneal transplantation in the Western world, the concurrent shortage of corneal transplants underscores the urgent need for further research into the underlying mechanisms. Such investigations could enable the development of innovative therapeutic strategies. Therefore, we aimed to verify candidate genes previously identified and sought after novel variants in the German population. Undertaking a genome wide association study (GWAS) using the Axiom™ Precision Medicine Diversity Array on 157 FECD cases and 309 controls, followed by pathway enrichment analysis, we were able to confirm the significance of the *TCF4* locus (rs613872, *p* = 8.0 × 10^− 23^, OR = 8.60, h^2^ = 0.72) and identified a range of novel variants. Further fine-mapping highlighted novel candidate SNPs, such as on chromosome 5 in the *SEMA6A* gene (rs153643, *p* = 3.1 × 10^− 9^, OR = 2.75, h^2^ = 0.30), and on chromosome 19 in the *DNAJC19P3* gene (rs62117964, *p* = 3.3 × 10^− 8^, OR = 3.61, h^2^ = 0.29). *SEMA6A* gene is involved in apoptotic pathways and cytoskeletal remodeling, making it an interesting candidate gene for further investigations as a potential therapeutic target. Furthermore, several variants were identified in lncRNAs, which presumably influence the expression of nearby protein-coding genes. For example, *LOC105372130*, which is associated with corneal hysteresis and corneal resistance factor, may influence the expression of *TCF4*.

**Supplementary Information:**

The online version contains supplementary material available at 10.1007/s00439-025-02749-7.

## Introduction

Fuchs’ Endothelial Corneal Dystrophy (FECD) was first reported in 1910 by Ernst Fuchs, termed “dystrophia epithelialis” (Fuchs 1910). It was later renamed, and it is nowadays recognized as one of the most common corneal diseases. This bilateral and slowly progressive disorder of the corneal endothelium and Descement’s membrane can lead to severe visual impairment, predominantly affecting women, with an up to four times higher prevalence than in men. In the majority of the cases, symptoms manifest after the age of 50. The prevalence of FECD varies globally, ranging from 3,7% in Japan and 4% in the USA, to 9,1% in Iceland (Ong Tone et al. 2021). Although the exact prevalence of FECD in Germany remains unknown, it is the leading cause of keratoplasties, accounting for 46% of cases (Flockerzi et al. 2018).

The substantial loss of non-regenerable endothelial cells and the subsequent decline in the ability to drain the corneal stroma, along with the accumulation of extracellular matrix leading to thickening and excrescences of the Descemet’s membrane (so-called guttae), result in irregularities of the inner corneal layers and stromal collagens. Ultimately, this dysfunction leads to progressive corneal edema with a subsequent opacification of the cornea and epithelial bullae. The affected patients suffer from glare, vision loss, and - in advanced cases - pain, ulcerations, and corneal fibrosis.

Conservative therapeutical management, e.g. including hyperosmolar eye drops, often proves insufficient. While perforating keratoplasty was the only surgical option for many years, its invasiveness often led to intervention at a late stage of the disease. There has been a notable shift towards posterior lamellar keratoplastic surgery, such as Descemet Membrane Endothelial Keratoplasty (DMEK) and Descemet Stripping Automated Endothelial Keratoplasty (DSAEK) (Brockmann et al. 2015). These procedures, known for their minimal invasiveness and short convalescence, are now usually indicated at an earlier stage of the disease (Gain et al. 2016; Flockerzi et al. 2018; Bachmann et al. 2019; Brockmann et al. 2018, 2019).

Since the late 1970s, familial clustering of FECD has been known, with Krachmer et al.(Krachmer et al. 1978) and Rosenblum et al.(Rosenblum et al. 1980) postulating an autosomal-dominant inheritance pattern. Subsequently, a series of genetic linkage and fine-mapping studies were performed. The much rarer early-onset form, typically symptomatic between 30 and 40 years of age, was found to be associated with genetic variants in the *COL8A2* gene on chromosome (chr.) 1 (Biswas 2001; Gottsch et al. 2005; Mok et al. 2009). Further genome-wide association studies (GWAS) identified variants in the genes *TCF4*, *SLC4A11*, *TCF8*, *KANK4*, *LAMC1*, *ATP1B1*, and *LOXHD1* to be associated with the late-onset form of FECD (Afshari et al. 2017; Baratz et al. 2010). In particular, the Single Nucleotide Polymorphism (SNP) rs613872 in the *TCF4* gene on chr. 18 has been extensively observed to be associated with FECD cases (Afshari et al. 2017; Baratz et al. 2010), together with an expansion of cytosine-thymine-guanine (CTG)_n_ repeat, known as CTG18.1 (Mootha et al. 2015; Foja et al. 2017; Xing et al. 2014; Fautsch et al. 2021; Westin et al. 2021; Wieben et al. 2012). Although observed in different populations, CTG18.1 shows the strongest association and highest frequency in European ancestry (Nanda et al. 2014; Eghrari et al. 2017; Peachey et al. 2023).

Besides the loci on chr. 18, the latest and largest multi-ancestry GWAS to date identified, by meta-analyzing with the previous largest GWAS, eight novel loci (Peachey et al. 2023): *SSBP3* (chr.1), *THSD7A* (chr.7), *LAMB1* (chr.7), *CDC151* and *PIDD1* (chr.11), *RORA* (chr.15), *HS3ST3B1* (chr.17), *LAMA5* (chr.20), and *COL18A1* (chr. 21). Together with the LAMC1-locus, *LAMA5* and *LAMB1* form the laminin-511 heterotrimer (LM511) and thus making this multi-functional adhesion protein a good candidate to be involved in the pathogenesis of FECD (Peachey et al. 2023).

Since the Central European population is becoming overall older, there will be a rising need for corneal transplants. Besides that, undergoing transplantation means the burden of long physical positioning as well as multiple daily applications of eyedrops for a rather long time - apart from the risk of surgical complications and graft rejection or failure (Brockmann et al. 2014).

In order to enhance the development of novel therapeutic strategies, our primary objective was to explore the genetic underpinnings of FECD within the German population. While there have been three investigations genotyping *TCF4* loci in German FECD patients, no GWAS was performed ( Okumura et al. 2019a, b; Foja et al. 2017). Addressing this deficiency, our research not only sought to verify previously associated loci but also to potentially uncover novel genetic contributors to FECD. This study benefits from an ethnically homogeneous cohort, thereby minimizing confounding genetic variability and enhancing the robustness of our findings. While previous studies have included individuals of European ancestry, none to date has focused exclusively on a German population. As all participants were recruited from the same clinical center, we infer not only a high degree of genetic homogeneity but also environmental consistency, particularly with regard to lifestyle and cultural factors.

A distinctive feature of our study is the application of the Axiom™ Precision Medicine Diversity Array, which offers broad coverage of clinically relevant genetic variants. To the best of our knowledge, this array has not previously been employed in genome-wide association studies (GWAS) on Fuchs endothelial corneal dystrophy (FECD), thus providing a unique opportunity to uncover novel SNPs associated with the disease.

## Materials and methods

### Samples and phenotypes

The study was performed in accordance with the Declaration of Helsinki and the protocol approved by the institutional ethical review committee of the University of Rostock (Ethics Application and Amendment A 2021-0048). Informed written consent was obtained from all subjects in the discovery and the control sample. The enrollment of subjects for both groups (discovery and control) was conducted in the Department of Ophthalmology of the Rostock University Medical Centre during clinics and entailed detailed ophthalmic examination including specular biomicroscopy and funduscopy. Discovery samples were considered to have FECD if corneal guttae with, or without corneal edema were present in both eyes or, if the patient had undergone Descemet Membrane Endothelial Keratoplasty or penetrating keratoplastic surgery in at least one eye because of bilateral corneal guttae.

Control subjects were enrolled from patients of the Department of Ophthalmology with a safe exclusion of corneal guttae and stromal or epithelial corneal edema related to corneal diseases. Nearly all individuals reported Central European descendance (13 not stated, 11 East-European/Russian/Kazakh) and all participants were unrelated individuals. The final dataset comprised 157 primary FECD cases and 309 controls.

### Genomic data

DNA extraction was carried out on whole blood using the Axiom™ 2.0 Plus Reagent Kit 96 F. Subsequently, the 466 samples underwent genotyping with the Axiom™ Precision Medicine Diversity Array, comprising a total of direct 868,337 markers, including 823,505 SNPs and 44,832 indels. The determination of chromosome, position, and reference and alternative alleles was based on the human GRCh38.p14 reference genome version.

Markers with a Minor Allele Frequency (MAF) < 0.05, a call rate < 90%, or those that failed the Hardy-Weinberg exact test were systematically excluded. In addition, only markers with at least five observed genotypes per genotype group (AA, AB, and BB) were considered. Ultimately, a selection of 247,015 markers were used for further analysis.

### Statistical analysis

A logistic regression analysis was conducted using PLINK v2.00a4 (Purcell et al. 2007) with the *-glm* function. Sex chromosomes were also included in analysis, being X coded as diploid for both females and males (*--xchr-model 2*), taking into account the human pseudo-autosomal regions (*--split-par hg38*), and Y coded as haploid. Principal Component Analysis (PCA), estimated with sklearn v1.3.0 library in Python, revealed no evident population stratification either within or between FECD cases and controls (Supplementary Fig. 1). Sex and age at diagnosis or age at the time of inclusion in the study were considered as covariates in the model as they showed an improved Akaike Information Criterion (AIC) in comparison to a null model without any covariates. Adding the first principal component (PC) to the model did not show any improvement in AIC, and was, therefore, not considered. Significant associations with FECD were identified for markers with a p-value below the threshold of 5 × 10^− 8^ (or exceeded -log_10_(p-value) = 7.3) considering multiple testing correction. The overall genomic inflation factor λ was estimated at 1.02 (Supplementary Fig. 2). For that reason, no adjustments were made for genomic control. Linkage disequilibrium between significant markers was estimated as R^2^ using PLINK v1.7. Additionally, statistical differences between genotype groups from the significant markers was compared by means of t-test for independent samples on the SciPy v1.10.1 library in Python.

A shotgun stochastic search approach was conducted for fine-mapping significant markers located within 100 Mb of each other to predict causal candidate variants using FINEMAP v1.4.2 (Benner et al. 2016).

### Pathway enrichment analysis

Genes comprising significant markers were annotated by Variant Effect Predictor, Ensembl release 110 (McLaren et al. 2016). Pathway enrichment analysis was performed for these genes using g: Profiler ve110_eg57_p18_4b54a898 (Raudvere et al. 2019).

### Genetic risk score

Genetic risk score per individual was calculated by multiplying the log(odds ratio) of each significant marker by the respective allele count for the individual, and summing these products over all significant markers, similarly to (Wray et al. 2018).

For estimating severity of the disease there was a classification into three groups – based on the more severely affected eye and under utilization of central corneal thickness measurements by the Pentacam (Oculus Pentacam HR) on the date of study inclusion:

Grade 1 (*n* = 36): guttae only; 

Grade 2 (*n* = 107): central corneal thickness > 600 μm, stromal edema, or clinically significant guttae;

Grade 3 (*n* = 14): decompensated disease with bullous keratopathy / epithelial edema;

based on (Yasukura et al. 2021). Patients already undergone keratoplasty were staged Grade 2 unless it was given in the patient’s history that the disease was decompensated. In that case they were classified Grade 3.

## Results

Our study group reflects the expected clinical gender and age distribution observed in the general population. Over 75% of our study group patients were 66 years or older, while all patients were 40 years or older, and either still suffered from FECD or underwent keratoplasty within the last years. The median age at the time of inclusion in the study was 72 years with a range of min. 40 and max. 91 years. This includes both patients who have undergone surgery and those who have not, with females comprising 62% of the cases. The control group is slightly younger, with a median age of 68 years and a range of min. 19 and max. 89 years. The gender distribution in this group is nearly equal, with males representing 49% and females 51%.

**Fig. 1:**
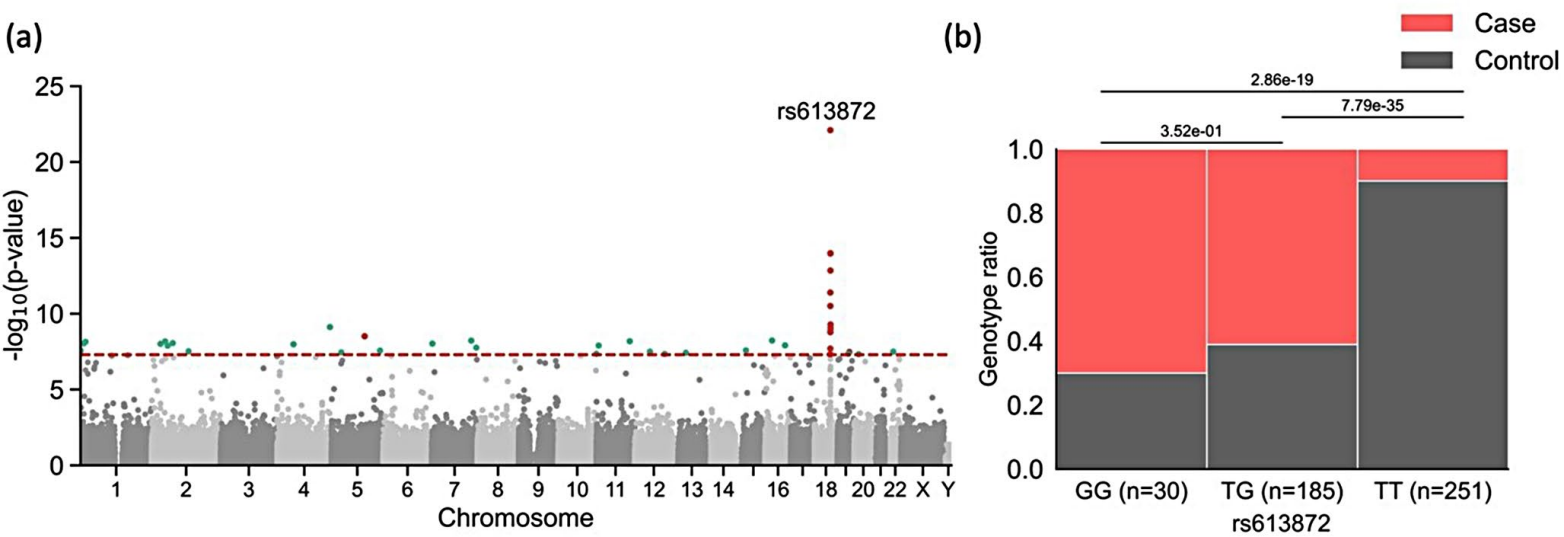
Genome-wide Association Study Results for FECD: (**a**) Manhattan plot of significant markers. The red line marks the significant threshold of 7.3. Red dots represent markers with an odds ratio > 1 while green represent dots with an odds ratio < 1. (**b**) Proportions of cases and controls in different genotype groups for the SNP rs613872

**Fig. 2:**
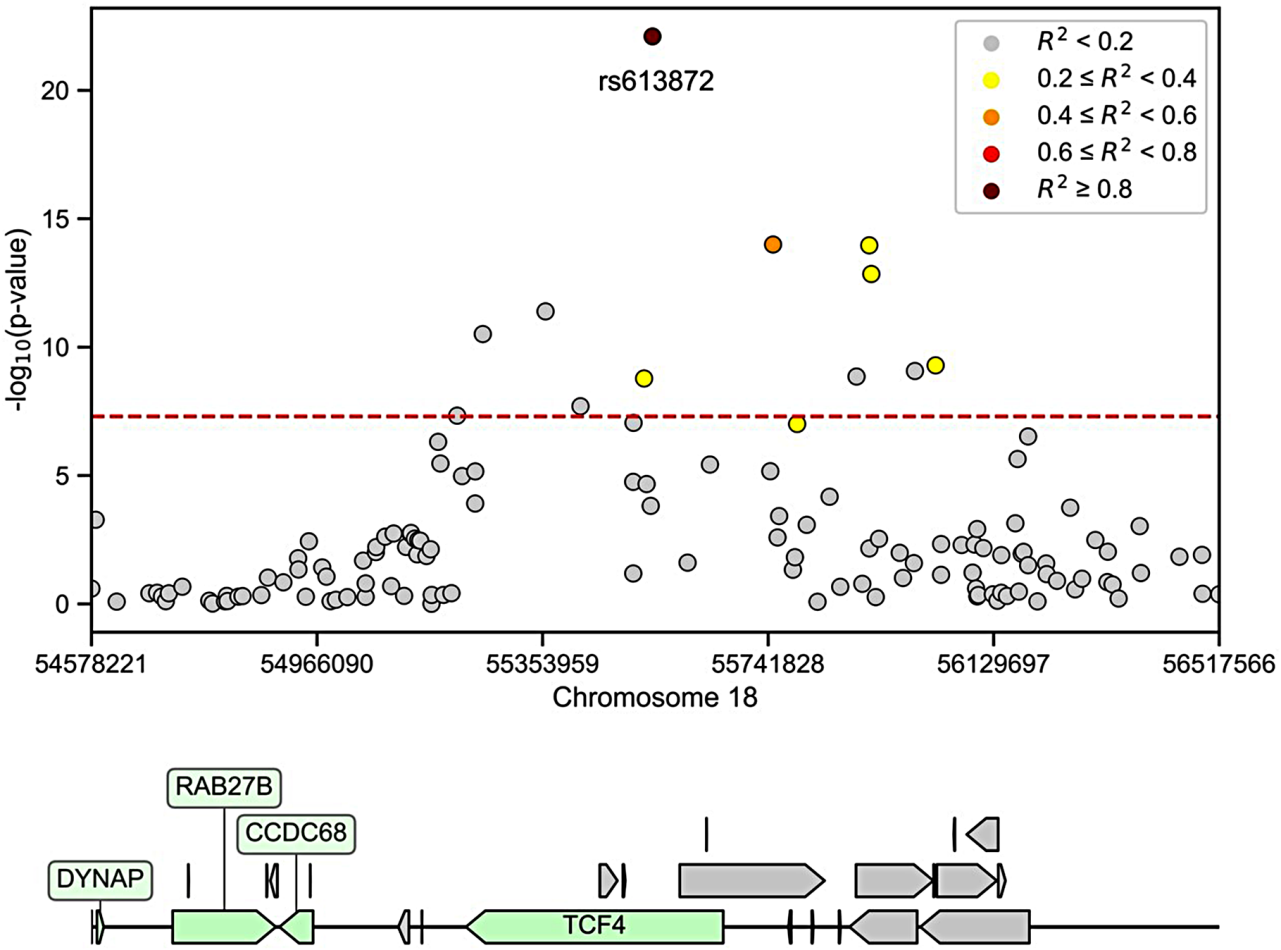
Linkage Disequilibrium between the Top Marker rs613872 and Neighboring Markers within 1 Mb Up and Downstream. The colors of the dots represent the level of linkage (R^2^), which can vary from 0 to 1. Genes spanning the region are shown at the bottom. Protein coding genes are represented in green, while long non-coding RNAs and pseudo-genes are shown in gray, including *ENSG00000267311*,* ENSG00000267284*,* MAP1LC3P*,* LINC01416*,* ENSG00000287944*,* ENSG00000267112*,* RPSAP57*,* ENSG00000267327*,* ENSG00000267732*,* LINC01905*,* LINC01539*,* ENSG00000267399*,* ENSG00000287248*,* LINC03069*,* SNORA73*,* LINC01929*,* RNA5SP459*,* TCF4-AS1*,* MIR4529*,* TCF4-AS2*,* RPL21P126*,* LINC01415*,* ENSG00000267172*, and *DYNAPP3*

The GWAS for FECD conducted on the German population uncovered a strong association within chr. 18, specifically in the region spanning from 54.5 Mb to 56.5 Mb, as illustrated in Fig. 1a. This region encompasses the well-established gene transcription factor 4 (*TCF4*) and other long non-protein coding RNAs (lncRNA) such as *LINC01416*, *LINC03069*, and *LOC105372130* (Table 1; Fig. 2).

Besides the association on chr. 18, two other markers on chromosomes 5 and 19 also showed a significant association to FECD with an odds ratio (OR) > 1, indicating an increased likelihood of developing FECD. Markers on chromosomes 1, 2, 4, 5, 7, 11, 12, 13, 15, 16, and 22 were also significant, but with an OR < 1, indicating a protective feature against FECD.

The strongest association was identified for the SNP rs613872, where the presence of allele G substantially raises the OR for developing FECD by 8.6-fold (Table 1). Interestingly, there was no statistically significant difference in the proportion of FECD cases between individuals with heterozygous TG and homozygous GG genotypes, as depicted in Fig. 1b. This suggests a dominant effect of allele G. The SNP rs613872 is situated within the third intron of the *TCF4* gene. As anticipated, a few other markers that exhibited a notable association with FECD display a certain degree of linkage disequilibrium with rs613872, as illustrated in Fig. 2.

**Table 1:** Markers significantly associated with FECD. Chr = chromosome, Ref = reference allele, Alt = alternative allele, MA = minor allele, MAF = minor allele frequency, OR = odds ratio, RSID = marker identifier

Chr	Pos	Ref	Alt	MA	MAF	OR	*P*-value	RSID	Genes
18	55,543,071	G	T	G	0.26	8.60	8.0 × 10^− 23^	rs613872	*TCF4*
18	55,750,408	A	G	A	0.24	4.06	1.0 × 10^− 14^	rs796743	*ENSG00000267284*,* LOC105372130*
18	55,915,845	T	TCC	TCC	0.25	3.97	1.1 × 10^− 14^	rs139805436	*LINC01416*,* ENSG00000267327*
19	41,638,282	G	A	A	0.11	3.61	3.3 × 10^− 08^	rs62117964	*DNAJC19P3*,* ENSG00000269354*,* LOC105372405*
18	55,919,359	G	A	A	0.26	3.59	1.4 × 10^− 13^	rs35248721	*LINC01416*,* ENSG00000267327*
18	55,994,381	T	C	C	0.17	3.33	8.5 × 10^− 10^	rs117550450	*ENSG00000267327*,* LINC01416*,* LOC107985164*
18	55,359,126	G	A	A	0.44	3.06	4.1 × 10^− 12^	rs1631486	*TCF4*
18	55,251,269	G	A	A	0.46	2.89	3.1 × 10^− 11^	rs1893431	*TCF4*
5	116,504,511	C	T	C	0.27	2.75	3.1 × 10^− 09^	rs153643	*SEMA6A*,* ENSG00000248445*
18	55,528,657	T	C	T	0.42	2.60	1.7 × 10^− 09^	rs4801158	*TCF4*
18	56,029,905	T	C	C	0.36	2.60	5.2 × 10^− 10^	rs764595	*LINC03069*,* ENSG00000287248*,* LINC01905*,* LOC642484*,* LOC107985164*
18	55,893,939	G	A	G	0.37	2.53	1.4 × 10^− 09^	rs9962249	*LINC01416*,* ENSG00000267327*
18	55,419,224	C	T	T	0.31	2.41	2.0 × 10^− 08^	rs1440477	*TCF4*
18	55,207,168	G	A	A	0.46	2.26	4.7 × 10^− 08^	rs8084537	
2	56,297,258	G	A	A	0.27	0.34	1.3 × 10^− 08^	rs62167331	*CCDC85A*
1	1,189,840	C	T	T	0.18	0.26	2.7 × 10^− 08^	rs12065129	*TTLL10*
16	24,576,889	C	A	A	0.20	0.26	5.9 × 10^− 09^	rs9933532	*RBBP6*
12	104,710,684	G	A	A	0.17	0.25	4.5 × 10^− 08^	rs61558853	*CHST11*
5	170,328,134	C	T	T	0.15	0.21	2.7 × 10^− 08^		*LINC01366*
4	184,858,106	C	T	T	0.18	0.21	7.7 × 10^− 10^	rs73873106	*MIR3945HG*,* ENSG00000289139*
2	31,305,043	G	A	A	0.16	0.20	9.7 × 10^− 09^	rs11124257	*LOC124907751*
11	117,204,850	C	T	T	0.16	0.20	6.6 × 10^− 09^	rs508487	*TAGLN*,* PCSK7*,* ENSG00000280143*,* LOC100652768*
11	8,227,467	G	C	C	0.15	0.20	1.3 × 10^− 08^	rs111887198	*LMO1*,* LOC105376536*
1	17,623,067	C	T	T	0.15	0.19	7.1 × 10^− 09^	rs55693639	*ARHGEF10L*
19	38,385,834	C	T	T	0.13	0.19	4.4 × 10^− 08^	rs183192050	*PSMD8*,* GGN*,* SPRED3*,* ENSG00000267090*
7	155,811,405	A	G	A	0.15	0.18	1.7 × 10^− 08^	rs756884	*SHH*
7	138,361,050	G	A	A	0.15	0.18	6.0 × 10^− 09^	rs79872067	
2	74,047,202	C	T	T	0.14	0.18	8.8 × 10^− 09^	rs61741171	*TET3*
22	26,031,882	C	T	T	0.13	0.18	3.2 × 10^− 08^	rs5752257	*MYO18B*
11	203,788	C	T	T	0.13	0.17	4.4 × 10^− 08^	rs2280543	*ODF3*,* BET1L*,* RIC8A*,* ENSG00000254559*
2	129,896,833	G	A	A	0.13	0.17	3.0 × 10^− 08^	rs10205980	*PLAC9P1*
12	53,079,461	TG	T	T	0.13	0.17	3.2 × 10^− 08^	rs28372672	*SPRYD3*
7	1,971,792	C	T	T	0.14	0.15	9.4 × 10^− 09^	rs117106123	*MAD1L1*
5	34,769,268	C	T	T	0.12	0.15	3.5 × 10^− 08^	rs115801393	*RAI14*
15	34,803,806	C	T	T	0.12	0.14	2.6 × 10^− 08^	rs56403318	*ENSG00000250007*,* LOC107984776*
16	70,157,320	G	A	A	0.12	0.14	1.2 × 10^− 08^		*PDPR*,* ENSG00000247228*,* LOC400541*
2	47,938,517	C	T	T	0.13	0.12	6.7 × 10^− 09^	rs62139957	*PPIAP62*,* ENSG00000230773*,* LOC105374591*,* LOC105374592*
20	15,743,996	G	A	A	0.12	0.11	4.7 × 10^− 08^	rs62194112	*MACROD2*
1	12,203,460	C	A	A	0.12	0.10	9.2 × 10^− 09^	rs17884378	*TNFRSF1B*
13	45,680,614	C	T	T	0.11	0.09	3.8 × 10^− 08^	rs113604798	*LINC01055*
4	57,405,854	C	A	A	0.12	0.08	1.0 × 10^− 08^	rs75611110	

For each of the quantitative trait loci identified, where more than one SNP was detected, the most likely causal variant was estimated (Table 2). Significant candidate variants included the SNPs rs153643 in the *SEMA6A* gene, rs613872 in the *TCF4* gene, and rs62117964 in the *DNAJC19P3* gene. In addition, the heritability (h^2^) was estimated for these candidate variants, with the highest heritability observed for rs613872, which explains 72% of the phenotypic variance.

**Table 2:** Potential causal variant per associated locus. RSID = Marker identifier; Z-score = standardized score; probability = posterior inclusion probability that a given SNP is causal for FECD based on bayesian statistics; Log_10_BF = Logarithm of Bayes factor. Log_10_BF greater than 2 suggests considerable evidence that the SNP is causal; h^2^ = heritability. Since h^2^ is calculated independently for each candidate marker, the sum of all heritabilities can exceed 1

RSID	Z-score	Probability	Log_10_BF	h^2^
rs12065129	1.09	0.39	0.10	0.00
rs62167331	1.79	0.34	0.30	0.00
rs153643	16.12	1.00	10.00	0.30
rs79872067	0.62	0.50	0.00	0.00
rs111887198	0.68	0.51	0.02	0.00
rs61558853	0.95	0.54	0.07	0.00
rs9933532	1.13	0.57	0.12	0.00
rs613872	39.32	1.00	11.04	0.72
rs62117964	15.529	1.00	10.00	0.29

**Fig. 3:**
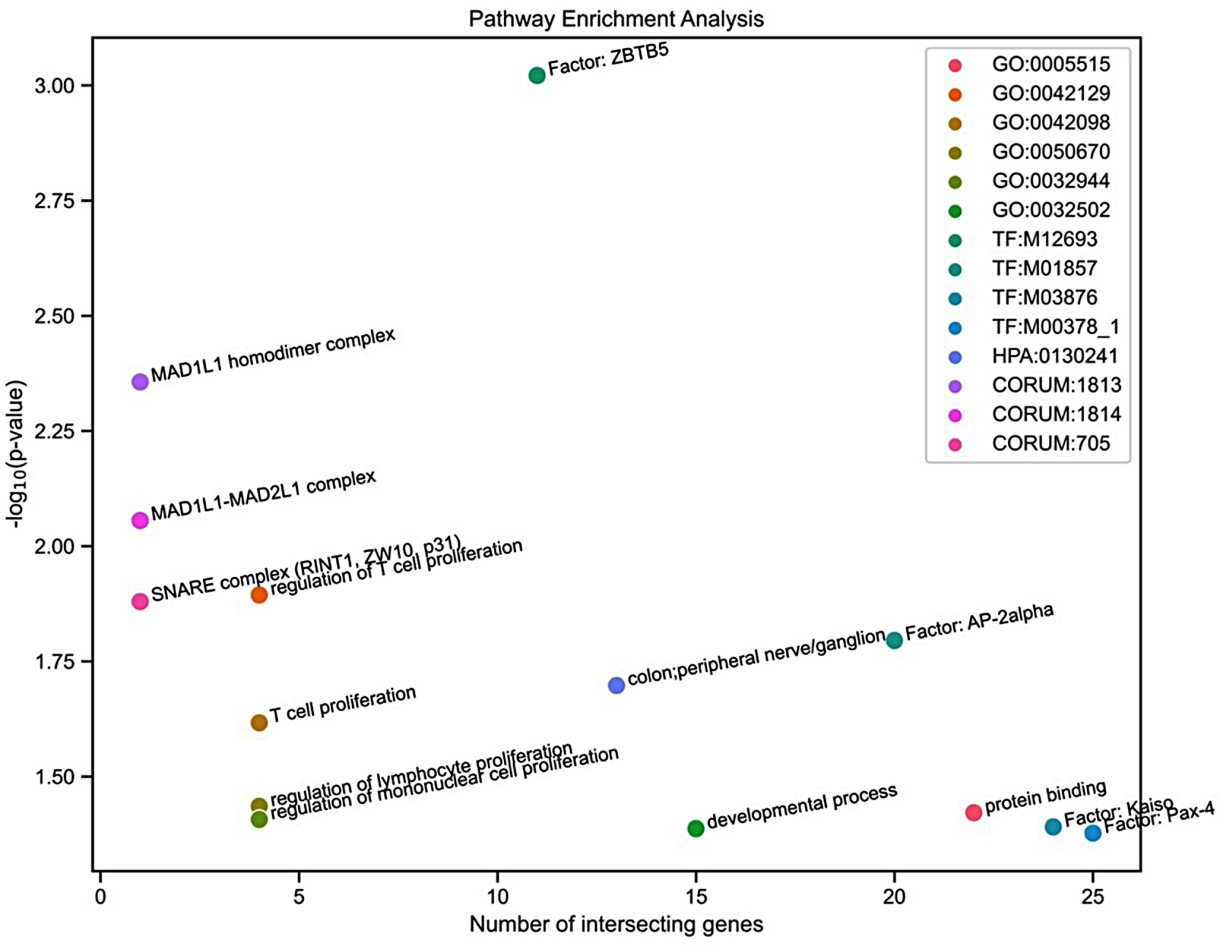
Pathway enrichment analysis for genes comprising markers significantly associated with FECD. Pathways come from different sources, namely Gene ontology (GO), TRANSFAC (TF), Human Protein Atlas (HPA), and CORUM

**Fig. 4:**
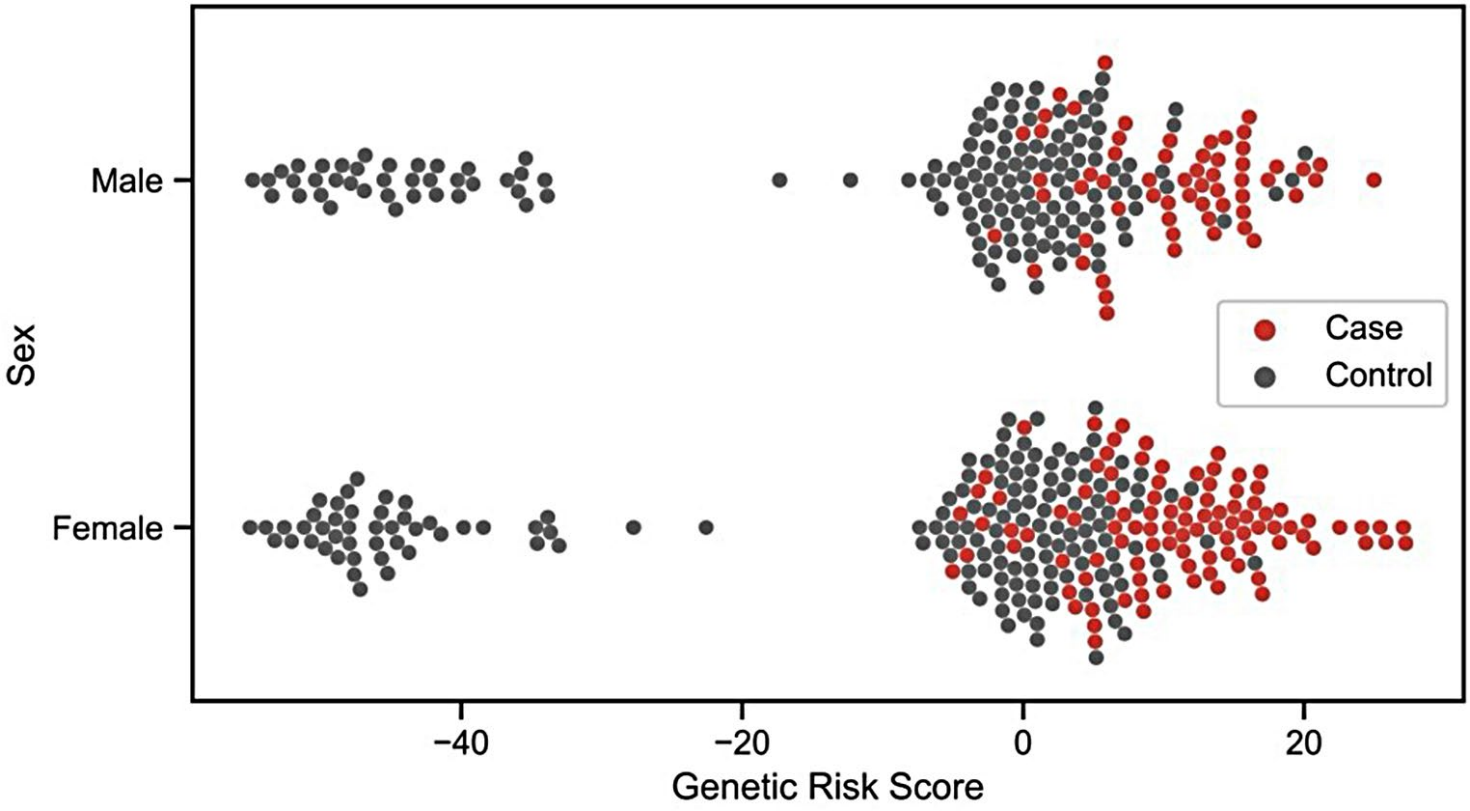
Genetic risk score estimation based on markers significantly associated with FECD

Genes comprising significant markers were enriched for diverse pathways, including GO terms for T cell proliferation and regulation of T cell, lymphocyte, and mononuclear cell proliferations. More general GO terms including developmental process and protein binding were also significantly enriched. Interestingly, the pathway from the Human Protein Atlas (HPA) (Thul and Lindskog 2018) for colon and peripheral nerve/ganglion was significant, together with some other enriched terms from TRANScription FACtor (TRANSFAC) and CORUM databases (Wingender et al. 2000; Tsitsiridis et al. 2023), including the transcription factors *ZBTB5* (zinc finger and BTB domain containing 5), *Kaiso* (zinc finger and BTB domain containing 33), *PAX-4* (paired box gene 4), and *AP-2alpha* (activating enhancer binding protein 2 alpha), and the MAD1L1 homodimer, MAD1L1-MAD2L, and SNARE (RINT1, ZW10, p31) complexes (Figs. 3, 4).

Genetic risk scores were estimated for all studied individuals based on all the markers’ OR significantly associated with FECD. Scores ranged from − 55 to 27. No clear differences were observed between male and female subjects. In general, the higher the score, the biggest the chances of developing FECD. Nevertheless, this configures just a predisposition, being, for instance, a control observed with a score of 20 and a case with a score of -5.

No statistical difference in the Genetic risk score was detected between the three severity groups. However, when only the *TCF4* locus (rs613872) was compared between the three severity groups, there was a significant difference (Dunn’s test p-value = 0.0006, after Bonferroni correction) between groups 1 and 2 (Supplementary Figures 3 and 4).

## Discussion

The current GWAS for FECD performed in the German population, predominantly comprising subjects of Central European ancestry, has succeeded in partially validating previously published results. Relevant known genetic associations are summarized in Supplementary Table 1. This validation is highlighted by confirming the significant relevance of chr. 18, particularly the region encompassing the *TCF4* locus. We were able to replicate the well-established SNP rs613872 (*p* = 8.0 × 10^− 23^, OR = 8.6) within the third intron of *TCF4*. We suggest that the allele G in rs613872 has a dominant effect, as initially observed by Krachmer et al. and Rosenblum et al. for FECD (Krachmer et al. 1978; Rosenblum et al. 1980).

Rs796743 in *LOC105372130* (*p* = 1.0 × 10^− 14^, OR = 4.06) is located near the previously identified lead SNP rs11659764 (Afshari et al. 2017; Peachey et al. 2023) which is not included in the current version of the Axiom™ Precision Medicine Diversity Array. *LOC105372130*, a lncRNA, is associated with corneal hysteresis (CH) and corneal resistance factor (CRF), intraocular pressure, and urinary albumin-to-creatinine ratio (UACR) (Simcoe et al. 2020; Khawaja et al. 2019; Choquet et al. 2017; Teumer et al. 2019). The association with biomechanical properties i.e. CH and CRF, which are both significantly lower in FECD than in normal eyes (del Buey et al. 2009), makes *LOC105372130* an interesting candidate gene for FECD in assumingly influencing the expression of *TCF4*.

*TCF4* is a protein coding gene for transcription factor 4, a basic helix-loop-helix TF which binds to E-Box, also known as E2-2 or FCD2. It is broadly expressed in more than 20 tissues and may play a key role in the nervous system development. Apart from the association with FECD it is associated with Pitt-Hopkins-Syndrome of mental retardation and facial abnormalities (Dean 2012). The locus rs1893431 is furthermore associated with less negative symptoms in schizophrenia (Wirgenes et al. 2012). Although the frequency of CTG18.1 TNR expansions in *TCF4* is not known for our samples, given its high linkage disequilibrium with rs613872 (Mootha et al. 2014) and previous studies in the German population (Foja et al. 2017), it can be assumed to be the primary causal variant for FECD.

*LINC01416* (rs9962249, rs139805436, rs35248721, rs117550450) is another lncRNA. Variants in LINC01416 are associated with phenotypes of central corneal thickness and the corneal resistance factor (Jiang et al. 2020; He et al. 2022). *LINC03069* (rs764595), also a lncRNA, among multiple phenotypes, is predominantly linked to behavior and schizophrenia, associated with central corneal thickness and corneal endothelial cell measurement (Ivarsdottir et al. 2019; He et al. 2022).

Being in moderate linkage disequilibrium with our lead-SNP rs613872 and significantly associated with FECD, the identified variants in lncRNAs lead to the assumption of (epigenetically) influencing the expression of *TCF4* in a way that is to be ascertained. LncRNAs have been associated with endothelial-to mesenchymal transition (EMT) in pulmonary fibrosis and proliferative vitreoretinopathy (Yildirim et al. 2021; Yang et al. 2021). While the process of EMT is part of the pathogenesis in FECD, lncRNA-mediated alteration in *TCF4*-expression might also have an influence on *ZEB1* and its induction of EMT.

Moreover, two genetic loci on chromosomes 19 and 5 with genome-wide significance and an OR > 1 were identified and fine-mapped as potential causal variants. First, rs153643 is located in an intron of the Semaphorin6A (*SEMA6A*) gene on chr. 5 (*p* = 3.1 × 10^− 09^, OR = 2,75). The gene *SEMA6A* is associated with the TUBBIII-expression and modification of actin-cytoskeleton by influencing the arrangement of both actin filaments and microtubule dynamics (Prislei et al. 2008; Yazdani and Terman 2006). As a class 6 semaphorin, the transmembrane molecule has the longest cytosolic domain and is one of 20 semaphorins in humans. Their receptors embrace the plexin family members, which feature a RasGTPase-activating protein domain with an inserted Rho GTPase-binding domain (Gurrapu and Tamagnone 2016). In zebrafish, SEMA6A is essential in conjunction with *PlexinA2* to maintain the integration of cells within the epithelium during eye (vesicle) development (Ebert et al. 2014). Cancer research brought out that the level of SEMA6A-protein expression has a prognostic significance in BRAF-mut melanoma patients by regulating the remodeling of actin cytoskeleton via inducing a SEMA6A-RhoA-YAP-pathway (Loria et al. 2022). Furthermore, it has a key function in cytosol-induced apoptosis in lung cancer cells (Shen et al. 2018). Despite the uncertain functional relevance of the observed significant intronic variant, these intriguing features of Semaphorin 6a are promising starting points for further investigations on its role in the development or promotion of FECD. Expression levels of Semaphorin 6a could be measured in corneal endothelial cells since both apoptosis and cytoskeletal remodeling are essential players in the disease. The variant rs62117964 located on chr. 19 is within *DNAJC19P3*, a pseudogene without any functions known so far.

There is a range of significant markers with an odds ratio below 1 suggesting a protective effect in the control group, somehow preventing it from developing FECD. One of these is rs55693639 in *ARHGEF10L* on chr 1. The gene encodes for Rho Guanine Nucleotide Exchange Factor 10 Like which belongs to RhoGEF (Guanine nucleotide Exchange Factor) subfamily of RhoGTPases. These are proteins involved in cell migration, adhesion and changes of actin cytoskeleton as well as microtubule dynamics. *ARHGEF10L* acts as a RhoGEF for RhoA, RhoB and RhoC, where RhoA may be required for corneal endothelial cell differentiation in mouse eyes (Mitchell D.C. et al. 2007) and is part of the p120-Kaiso-RhoA-ROCK signaling. Furthermore, (Ortega et al. 2016) observed that exposure to hyperosmotic stress resulted in enhanced actin polymerization and stimulated the activity of the Rho GTPases Rac1 and RhoA, while leaving Cdc42 unaffected. Inhibiting the pathways mediated by Rac1 and RhoA exerted a slight influence on the disruption of the barrier, yet it notably postponed the barrier’s restoration following the removal of stress. Conversely, the activation of Rac1 and RhoA not only bolstered the intrinsic function of the endothelial barrier but also expedited the repair process of the barrier. RhoA-ROCK signaling is coupled with canonical TGFβ-signaling. *TCF4* also participates in gene regulation (binding to E-box sequences of promoters and enhancers of specific genes) in the TGF-β and NF-κB signaling pathways, in mesenchymal transition, and apoptosis, contributing to endothelial cell loss in FECD.

In general, the variants with a protective feature associated with FECD may contribute to disease resistance by influencing metabolic, physiological, or behavioral pathways which indirectly decreases the chances of developing the disease.

Genes comprising significant markers were, among others, enriched for the pathway of *Kaiso* (zinc finger and BTB domain containing 33), a transcription factor that is involved in numerous biological processes such as epithelial-to-mesenchymal transition and apoptosis. It is playing a part in the expansion of human corneal endothelial cells (by p120-Kaiso-RhoA-ROCK signaling following knockdown of p120-Kaiso). It was shown that it is possible to reprogram HCECs into progenitor status using p120-Kaiso siRNA knockdown (Zhu et al. 2019; Y.-T. Zhu et al. 2012).

Furthermore, there was an enrichment for *AP-2alpha*, a member of the transcription factor of the Activating protein-2-family. It is involved in the differentiation of stratified epithelia including the ocular surface by inducing cadherin expression (West-Mays et al. 2003; Dwivedi et al. 2005).

*ZBTB5* (zinc finger and BTB domain containing 5) is a transcription factor and proto-oncogene that either leads to stimulation of cell proliferation when p53 is present or promotes apoptosis when p53 is absent (Choi et al. 2020). It was shown that *ZBTB5* expression is higher in retinoblastoma and muscle cancer tissues and is located in the nucleus. It is competing with p53 over the occupation of p53 binding elements and repressing the transcription of the cell cycle arrest gene p21 (Koh et al. 2009). Moreover, *ZBTB5* is involved in processes promoting cisplatin resistance in non-small cell lung cancer via a lncRNA (*LINC00221*) (Tang et al. 2019).

Another enriched term was MAD1L1, a component of the mitotic spindle-assembly checkpoint that prevents the onset of anaphase until all chromosomes are properly aligned at the metaphase plate. MAD1L1 functions as a homodimer and interacts with MAD2L1. MAD1L1 may play a role in cell cycle control and tumor suppression. Loss of MAD1 may lead to deficiencies in cellular attachment, adhesion, and FAK (focal adhesion kinase) actuation via disruption of α5 integrin secretion. Furthermore, correlating to the expression level, MAD1 hinders cell motility or alternatively expedites directed cell migration (Wan et al. 2014). Juang et al. (Juang 2011) identified that a lower expression level of E-Cadherin is a result of decreased MAD1 expression and leads to enhanced cell migration ability in breast-cancer cells. In our study a novel variant rs117106123 in MAD1L1 is significantly associated with FECD (OR 0.15; *P* = 9.4 × 10^− 09^). However, the variant is intronic and its consequence yet unclear. It is noteworthy that E-Cadherin emerges to be a key player in the enriched pathways from TRANSFAC and CORUM databases. Although Foja et al. did not detect E-Cadherin in any corneal endothelial cells of German patients, N-cadherin was detected both in controls and affected CECs (Foja et al. 2017). N-Cadherin is an inductor of endothelial to mesenchymal transition, which is part of the pathogenesis of FECD, and its expression in guttae-surrounding endothelial cells is raised (Kocaba et al. 2018).

Our GWAS analysis faces some limitations. The cohort size in our study is relatively small, similar to that of Baratz et al. (Baratz et al. 2010), but substantially less than those used by Afshari et al. and Peachey et al. (Afshari, Igo, et al. 2017; Peachey et al. 2023), potentially contributing to the detection of significant but dispersed SNPs without forming prominent association peaks outside of the *TCF4* locus. The Axiom™ Precision Medicine Diversity Array we utilized comprises 868,337 markers, with only a small overlap with the Illumina 370K and HumanOmni arrays (Verlouw et al. 2021). This overlap indicates a considerable, but not complete, coverage comparison with the arrays used in the aforementioned studies, which could impact the consistency and discovery of associated markers. To enhance the robustness of our findings, a replication study with an independent subject group would be prudent. Moreover, the use of imputation may increase the number of predicted SNPs without genotyping proximal variants, as previously employed in other studies. Additionally, the FECD cases in our study were not subjected to advanced staging using the Krachmer scale, nor were the diagnoses of those who underwent keratoplasty histo-pathologically confirmed, leaving a slim but present risk of misdiagnosis.

## Conclusion

We intended to set the identified novel loci into a suitable biological context to reveal their significance for the ongoing exploration of the genetic background and potential therapeutic targets of FECD. In our GWAS conducted on the German population using the Axiom™ Precision Medicine Diversity Array we could verify the previously identified *TCF4* locus and in particular the lead SNP rs613872 being highly associated with the development of FECD. Several dispersed SNPs occurred to be genome-wide significant, but lacked forming association peaks. These were comprising lncRNAs in moderate linkage disequilibrium to *TCF4* thus, corroborating the outstanding relevance of chromosome 18 in this context and bringing lncRNAs into focus of having a bearing on the pathogenesis. Moreover, the genome-wide significant variant in the *SEMA6A* gene as being involved in arrangement of actin filaments and microtubule dynamics as well as in apoptotic pathways presents opportunities for further investigations.

## Electronic supplementary material

Below is the link to the electronic supplementary material.

Supplementary Material 1

Supplementary Material 2

## Data Availability

The data that support the findings of this study are not openly available due to reasons of sensitivity and are available from the corresponding author upon reasonable request.
