# Supplementary material for: Genome-wide association study of Fuchs’ endothelial corneal dystrophy in the German population
Source: Hum Genet. 2025 May 12;144(6):653–64. doi: 10.1007/s00439-025-02749-7 (PMC12170761; doi:10.1007/s00439-025-02749-7)
Supplement: Supplementary file 1 — Supplementary Material 1 [file 439_2025_2749_MOESM1_ESM.docx]

**Supplementary Materials**


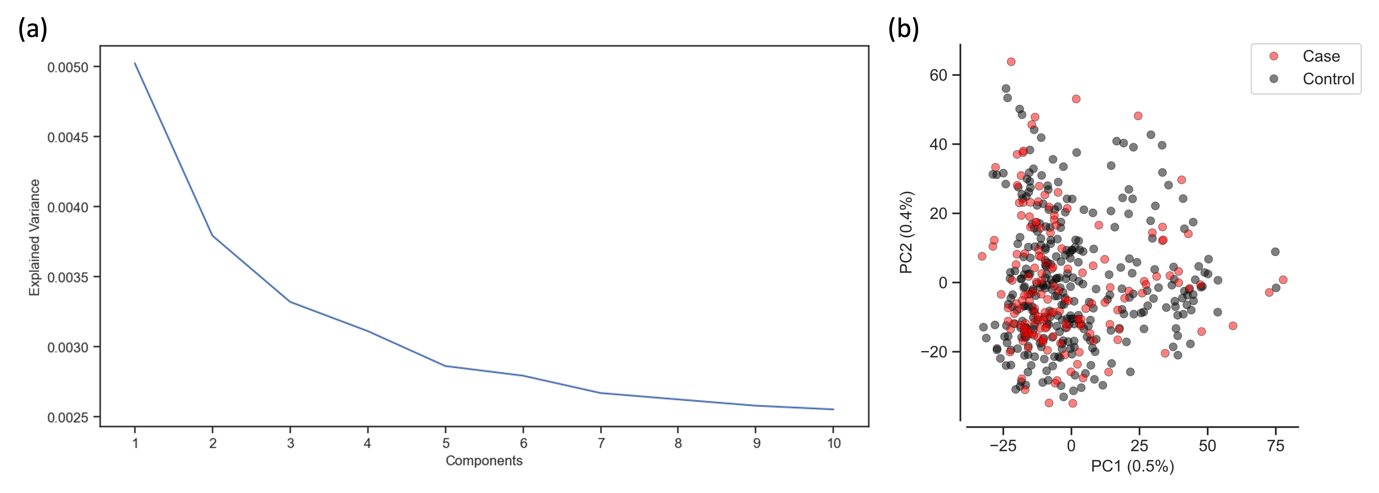


**Supplementary Figure 1 - PCA.** (a) Explained variability for the first ten principal components based on autosomal genotypes. (b) Eigenvalues for principal component 1 (PC1) and principal component 2 (PC2) for case and control samples.


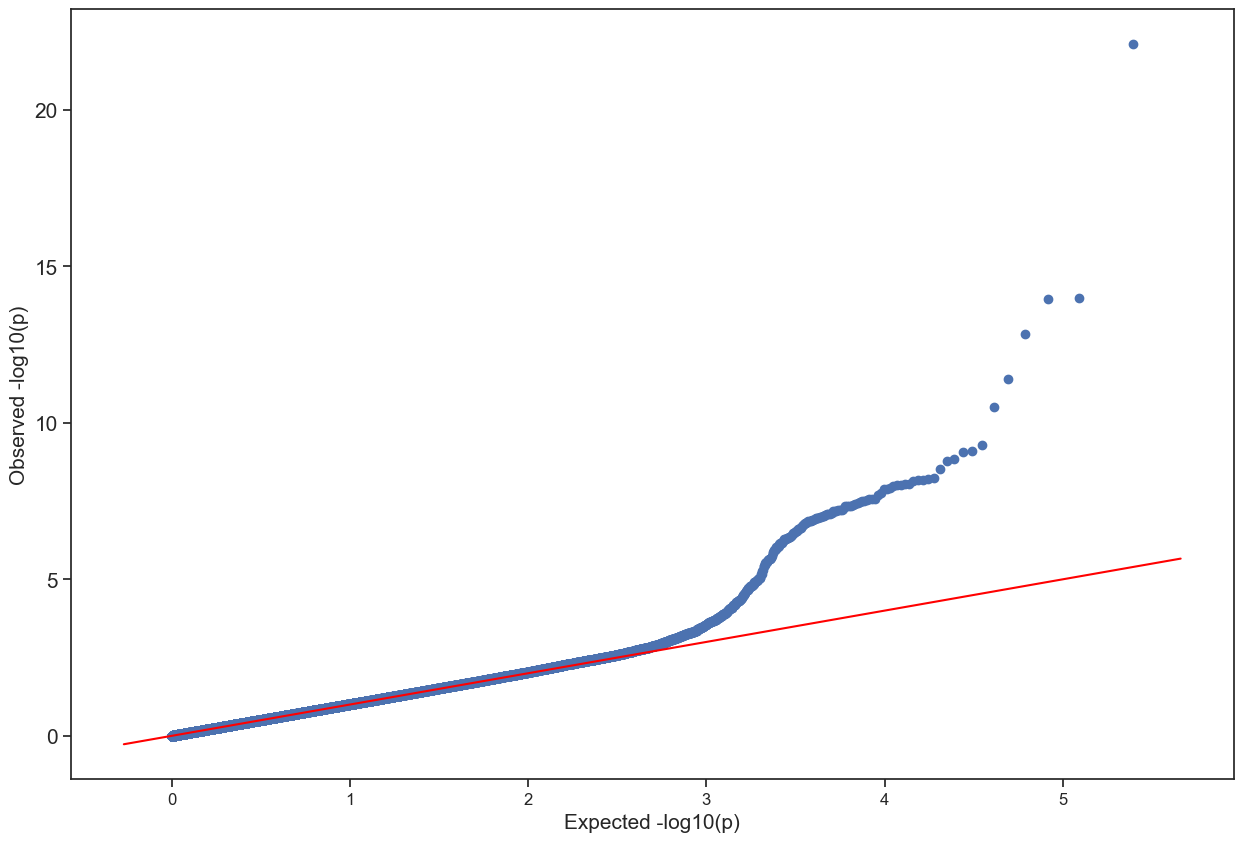


**Supplementary Figure 2 - QQPlot between expected and observed p-values**

**
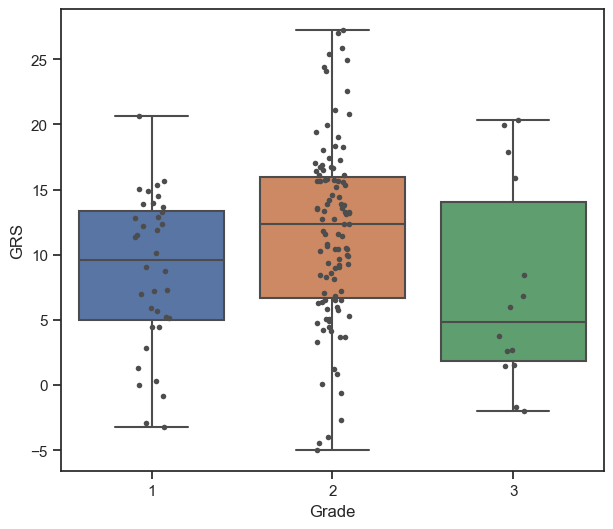
Supplementary Figure 3 - Differences in the Genetic Risk Score between Severity Grade**

**
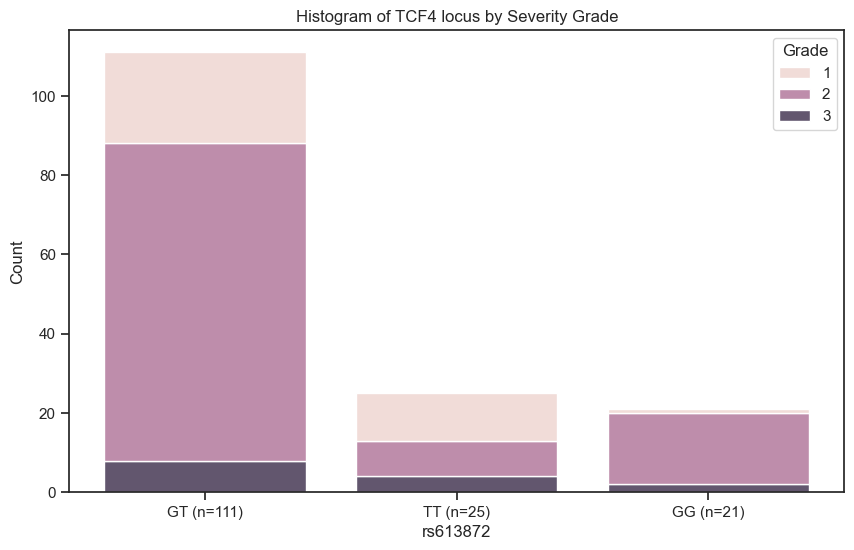
Supplementary Figure 4 – Histogram of TCF4 locus by Severity Grade**
